# Geopolitics and sports

**DOI:** 10.3389/fspor.2025.1650791

**Published:** 2025-09-04

**Authors:** Marko Begović, Simon Chadwick

**Affiliations:** ^1^ Faculty of Business Administration and Social Sciences, Molde University College, Molde, Norway; ^2^ Emlyon, Shanghai, China

**Keywords:** geopolitics, Global South, Global North, sportswashing, political neutrality, soft power

## Introduction

Following the capture of Goma—the biggest city in eastern DR Congo—by M23 rebels, the country's foreign minister (Thérèse Kayikwamba Wagner) reportedly wrote to the owners of European football clubs Arsenal, Bayern Munich, and Paris Saint-Germain. Each of the clubs is sponsored by the Visit Rwanda tourism campaign, in what some have labeled as “blood-stained” deals. In the first 6 months of 2025, an estimated 400,000 people were displaced by the M23, a group widely believed to be under the *de facto* control of Rwanda's government. Kayikwamba Wagner reportedly called the shirt sponsorship deals immoral. In the summer of 2025, Bayern terminated its deal with Visit Rwanda.

This is just one instance in a growing number of cases where sports and politics mix, albeit with links to business, countries, demography, cultures, ideologies, and so forth (1, 2). This might be an inconvenient reality for those who claim that sports and politics should not mix. However, the two are inextricably linked, resulting in sports having to operate in often complex and sensitive environments. This necessitates managers, leaders, and decision makers, as well as others with a stake in the industry, needing to have a strong, well-informed sense of the challenges they face (3). It is in this context that we have compiled a special geopolitics edition of this journal.

## Sports in a shifting world order

The interplay between politics and sports has evolved into a complex geopolitical dynamic, reflecting, from the perspective of the sports movement, a selective adherence to the International Olympic Committee's principle of political neutrality outlined in the Olympic Charter (4). The concept is embedded in the Charter as one of the fundamental principles of Olympism within the constitutional framework aimed at isolating sports from political (or any other outsider) interference. Moreover, the Charter mandates the IOC to take appropriate action to maintain this concept of the Olympic Movement, shaped by the culture of self-governance. Despite this framework aimed at opposing the politicization of sports and safeguarding autonomy, the principle remained increasingly contested through instrumentalization and growing governmentalization of sports for various non-sporting objectives. In parallel to contradicting the mission and purpose, it exposes the fragility of institutional and organizational autonomy of the sports movement, as the concept of political neutrality is subjected to geopolitical realities.

One of the biggest challenges facing sports is the fitness for the purpose of its institutions and structures, particularly from a hegemonic perspective (5). Many of its biggest current governing bodies were the outcome of significant European influence, the likes of FIFA and the IOC having been created by Europeans. Even today, their headquarters remain located in Europe, and, for much of their collective history, most of them have been presided over by Europeans. However, over the last 50 years, American influence on sports, primarily though not exclusively commercial, has grown. Over the last 30 years, sports have therefore been through a process of industrialization and commercialization, driven by a liberal free market ideology. More recently, globalization, digitalization, and climate change have resulted in the emergence of nations in the Global South as powerful members of the international sports community.^1^ As a result, this juxtaposition of often competing ideologies has created all manner of challenges, ranging from how to manage sponsorship deals (6) through to how sports should be governed, by whom, and according to what rules (1). Our intention is that this special edition helps bring some clarity to the way in which those working in sports view these challenges.

In a fast-changing world that is having profound implications for sports, the need for a better understanding of geopolitics is growing. Yet in the main, academic research has thus far failed to keep pace with the challenges sports now face. The breadth and depth of available literature are questionable, while the absence of multidisciplinary studies in published articles is stark (5). Such is the interconnected, networked nature of global sports that work drawing from several disciplines seems imperative. Moreover, studies undertaken from outside Western hegemony are vital; not only would this help bring diversity of perspective, it would also frame issues in ways that are more relevant to the conceptions and experiences of sports now evident in and emanating from the Global South. In calling for such new approaches to sports research, we equally acknowledge that new methodologies may be required to underpin new research studies that are now beginning to emerge.

## The tardy response of the academic community

The academic response has been equally ambiguous with the introduction of the sportswashing concept, disregarding the existence of propaganda through sports (or simply sports propaganda) from the emergence of contemporary sports in the 19th century, the establishment of international sports organizations, and the organization of major sports events. It is not a new phenomenon that states seek global legitimacy or undertake nation branding through sports; however, it means they are just refined in the approach and means to an end. Following WWII, globalization, along with the commercialization and professionalization of sports, redefined the importance of medal counts and podiums with the monetization of sports and tailoring soft power approach. From a bipolar battleground for ideological supremacy between the East and the West including mutual boycotts (1980 and 1984), the geopolitical shifts from unipolarity to multilateralism enabled asymmetric and emerging powers—including China, Qatar, Saudi Arabia, and the United Arab Emirates—to play a more prominent role in shaping, directing, and governing global sports (4). In parallel, the re-centralization or prioritization of sports as a public policy tool represents a reflex on the previously discussed dynamics, especially as an attempt to utilize the expansion of multinational corporations (including media conglomerates) and proliferation of international sporting events.

## Organizational hypocrisy

Naturally, the frictions and tensions reflect rather a non-sporting constellation that further erodes political neutrality as the bedrock of Olympism and the global sports movement, particularly in their challenge to steer the process of expanding the stakeholder network in the form of non-state actors, organizers of events, initiatives for new sports or related activities, hybrid actors, and quasi-governmental entities. That said, applied selectively with the degree of discretionary power, the concept of political neutrality lost its primary function as a guardian from political interference.

## Discussion

We acknowledge the lack of research interest in discussing the rather transformation of sports governance to transactional logic shaped by ambiguity, selective application of rules, and strategic opportunism. While few authors have been keen to picture these dynamics as the rise of illiberal actors in the form of sportswashing, most of them have remained silent in acknowledging the boomerang effect and disconnect between law-on-the-books and law-in-practice, where informal networks and transactional relations shape institutional outcomes. The International Tennis Federation's (ITF) limited reaction to the Lawn Tennis Association's (LTA) and Wimbledon's organizer's decision to ban Russian and Belarussian tennis players in 2022, and FIFA/UEFA's institutional ambiguity on the Israel Football Association (IFA) breach of their own statutory provisions, confirm institutional contradictions in undermining one of the key principles and mimicking or coercing to dominant geopolitical narratives (7).

We conclude that while sports are currently operating in complex and sensitive conditions, where risk and security are major challenges, this is nevertheless one of the most dynamic, exciting periods in the sector's history. This demands that academic research step into new territories, creating new perspectives, developing new methodologies, and bringing new insights into a world that is pivoting from the Global North to the Global South. We feel that this special edition of the journal makes a contribution to a new field of work that links sports with geopolitics and beyond.

## Footnotes

^1^The terms ““Global North”“ and ““Global South”“ reflect historical, geopolitical, and economic divisions. The Global South is associated with countries located in Africa, Asia, and Latin America, including parts of the Middle East, e.g., Morocco, India, Paraguay, and Libya. The Global North consists of the most developed economic and high-income countries, e.g., the United States of America, Canada, Norway, Japan, and Australia.
